# Supplementary material for: Neural sensitization improves encoding fidelity in the primate retina
Source: Nat Commun. 2019 Sep 5;10:4017. doi: 10.1038/s41467-019-11734-4 (PMC6728337; doi:10.1038/s41467-019-11734-4)

Supplementary Information

Neural sensitization improves encoding fidelity in the primate retina

Appleby and Manookin

Supplementary Figures

Supplementary Figure 1. Time course of contrast sensitization and adaptation. a Change in spike rate for the adapted condition relative to unadapted control for adaptation periods (contrast, ±0.25–0.5; delay 0.05 s). Adaptation period was varied between 0.25–1.25 s (*x*-axis). The adapting stimulus produced a significant increase in spiking for each of the durations tested ($p < 4.0 \times 10^{-3}$; n = 9 cells). **b** Duration of contrast sensitization in midget ganglion cells. Test flashes (contrast, ±0.25–0.5) were presented at different delays (*x*-axis) following the offset of an adapting stimulus. Percent change in spike rate for the adapted condition relative to the unadapted condition is shown on the *y*-axis. Increase in spiking was statistically significant for delays ≤0.4 s ($p < 7.0 \times 10^{-3}$; n = 11 cells). **c** Same as **b** for parasol ganglion cells. The adapting stimulus significantly reduced spiking at delays ≤0.8 s ($p < 7.0 \times 10^{-3}$; n = 10 cells). Error bars indicate mean ± SEM. Statistical analyses were paired, and significance was calculated using the Wilcoxon signed rank test.

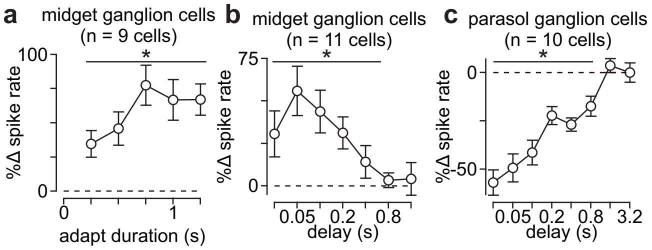

Supplementary Figure 2. Sensitization model reproduces experimental results. **a** Sensitization model structure. Visual inputs were convolved with a spatiotemporal linear filter comprised of a Gaussian in space and a biphasic filter in time. Signals in the amacrine cell pathway were then passed through an output nonlinearity before passing to the adaptation stage of the model. The output of the amacrine cell model provided inhibitory input to the midget bipolar cell pathway upstream of the bipolar cell output nonlinearity. **b** Inhibitory temporal filter (*left*) and input-output nonlinearity (*right*) determined from noise recordings. These filters were then used as components of the computational model **a**. **c** Excitatory current recording from an Off midget ganglion cell to the wide-field adapting stimulus (see Fig. 6). Model prediction (*orange*) was generated from excitatory synaptic current recordings to the noise stimulus in the same cell. **d** Model output for drifting grating stimuli at high and low spatial frequencies.

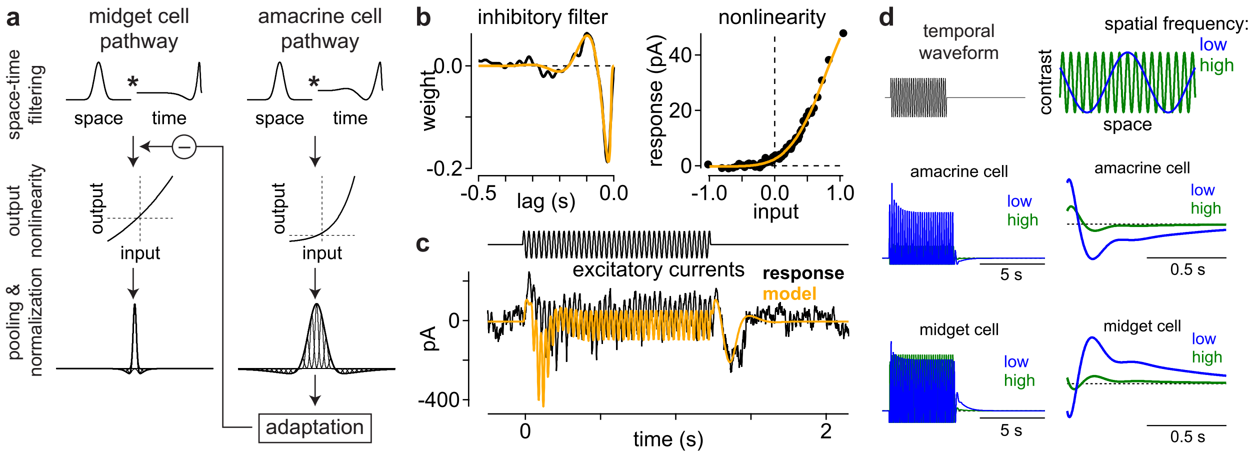

Supplement: Supplementary file 1 — Supplementary Information [file 41467_2019_11734_MOESM1_ESM.pdf]
