# Supplementary material for: Neural sensitization improves encoding fidelity in the primate retina
Source: Nat Commun. 2019 Sep 5;10:4017. doi: 10.1038/s41467-019-11734-4 (PMC6728337; doi:10.1038/s41467-019-11734-4)
Supplement: Supplementary file 3 — Reporting Summary [file 41467_2019_11734_MOESM3_ESM.pdf]

nature research

Corresponding author(s): Michael B. Manookin

Last updated by author(s): Jul 16, 2019

Reporting Summary

Nature Research wishes to improve the reproducibility of the work that we publish. This form provides structure for consistency and transparency in reporting. For further information on Nature Research policies, see Authors & Referees and the Editorial Policy Checklist.

Statistics

For all statistical analyses, confirm that the following items are present in the figure legend, table legend, main text, or Methods section.

n/a	Confirmed	
☐	☒	The exact sample size (n) for each experimental group/condition, given as a discrete number and unit of measurement
☐	☒	A statement on whether measurements were taken from distinct samples or whether the same sample was measured repeatedly
☐	☒	The statistical test(s) used AND whether they are one- or two-sided<br>*Only common tests should be described solely by name; describe more complex techniques in the Methods section.*
☐	☒	A description of all covariates tested
☒	☐	A description of any assumptions or corrections, such as tests of normality and adjustment for multiple comparisons
☐	☒	A full description of the statistical parameters including central tendency (e.g. means) or other basic estimates (e.g. regression coefficient) AND variation (e.g. standard deviation) or associated estimates of uncertainty (e.g. confidence intervals)
☐	☒	For null hypothesis testing, the test statistic (e.g. F, t, r) with confidence intervals, effect sizes, degrees of freedom and P value noted<br>*Give P values as exact values whenever suitable.*
☒	☐	For Bayesian analysis, information on the choice of priors and Markov chain Monte Carlo settings
☒	☐	For hierarchical and complex designs, identification of the appropriate level for tests and full reporting of outcomes
☐	☒	Estimates of effect sizes (e.g. Cohen's d, Pearson's r), indicating how they were calculated

Our web collection on statistics for biologists contains articles on many of the points above.

Software and code

Policy information about availability of computer code

Data collection	Visual stimulation (http://stage-vss.github.io) and data acquisition (http://symphony-das.github.io) software are freely available.
Data analysis	All statistical analyses were performed in MATLAB (R2018b, Mathworks).

For manuscripts utilizing custom algorithms or software that are central to the research but not yet described in published literature, software must be made available to editors/reviewers. We strongly encourage code deposition in a community repository (e.g. GitHub). See the Nature Research guidelines for submitting code & software for further information.

Data

Policy information about availability of data

All manuscripts must include a data availability statement. This statement should provide the following information, where applicable:

- Accession codes, unique identifiers, or web links for publicly available datasets
- A list of figures that have associated raw data
- A description of any restrictions on data availability

The datasets generated during and/or analysed during the current study are available from the corresponding author on reasonable request.

Field-specific reporting

Please select the one below that is the best fit for your research. If you are not sure, read the appropriate sections before making your selection.

☒ Life sciences ☐ Behavioural & social sciences ☐ Ecological, evolutionary & environmental sciences

For a reference copy of the document with all sections, see nature.com/documents/nr-reporting-summary-flat.pdf

Life sciences study design

All studies must disclose on these points even when the disclosure is negative.

Sample size	Sample size was determined from the cell count for each stimulus condition/experiment.
Data exclusions	Cell quality was determined by recording from parasol ganglion cells neighboring targeted midget cells. Parasol cells were expected to increase their spike rate by at least 20 spikes/s to a temporally modulated 5% contrast spot presented over the receptive field at a photopic mean luminance. Cells recorded in retinal regions that did not meet this selection criterion were excluded from the manuscript.
Replication	Each experiment was run on multiple cells of each type and appropriate statistical measures were employed.
Randomization	Stimulus conditions were randomly interleaved on each trial to decrease potential variability in the measurements.
Blinding	This is not applicable to physiology studies of this type.

Reporting for specific materials, systems and methods

We require information from authors about some types of materials, experimental systems and methods used in many studies. Here, indicate whether each material, system or method listed is relevant to your study. If you are not sure if a list item applies to your research, read the appropriate section before selecting a response.

Materials & experimental systems

n/a	Involved in the study	
☒	☐	Antibodies
☒	☐	Eukaryotic cell lines
☒	☐	Palaeontology
☐	☒	Animals and other organisms
☒	☐	Human research participants
☒	☐	Clinical data

Methods

n/a	Involved in the study	
☒	☐	ChIP-seq
☒	☐	Flow cytometry
☒	☐	MRI-based neuroimaging

Animals and other organisms

Policy information about studies involving animals; ARRIVE guidelines recommended for reporting animal research

Laboratory animals	Eyes were dissected from terminally anesthetized macaque monkeys of either sex (Macaca fascicularis, mulatta, and nemestrina) obtained through the Tissue Distribution Program of the National Primate Research Center at the University of Washington.
Wild animals	Wild animals were not involved in this study.
Field-collected samples	This study did not involved samples collected from the field.
Ethics oversight	All procedures were approved by the University of Washington Institutional Animal Care and Use Committee.

Note that full information on the approval of the study protocol must also be provided in the manuscript.
